# Left with a Sisyphean task – the experiences of nurse practitioners with treating non-suicidal self-injury in the emergency department: a descriptive qualitative study

**DOI:** 10.1186/s12873-023-00888-6

**Published:** 2023-10-05

**Authors:** Kickan Roed, Cecilie Rostrup Brauner, Senayt Yigzaw, Julie Midtgaard

**Affiliations:** 1 Mental Health Center Glostrup, Centre for Applied Research in Mental Health Care, Copenhagen University Hospital – Mental Health Services CPH, Nordstjernevej 41, Glostrup, 2600 Denmark; 2 Department of Clinical Medicine, Faculty of Health and Medical Sciences, University of Copenhagen, Blegdamsvej 3B, Copenhagen N, 2200 Denmark

**Keywords:** Non-suicidal self-injury, Nursing, Emergency department, Experience, Qualitative content analysis

## Abstract

**Background:**

Non-suicidal self-injury (NSSI) is a prevalent phenomenon in somatic emergency departments, where nurses are the most consistent group of healthcare professionals who treat people with NSSI, which means they may affect the NSSI trajectory and help-seeking in the future. The objective of this study was to describe the experiences of nurse practitioners with treatment of people presenting with NSSI in the emergency department.

**Methods:**

Individual, semi-structured telephone interviews were conducted with seventeen purposefully recruited nurse practitioners from three emergency departments in the Capital Region of Denmark. Interview transcripts were analysed using inductive content analysis, as described by Graneheim and Lundman.

**Results:**

The analysis resulted in the formulation of three categories and 10 subcategories describing how nurse practitioners feel confident and competent in treating physical injuries due to NSSI but at the same time insecure about how to provide adequate care and engage in conversations about NSSI and mental wellbeing with people with NSSI. An overarching theme, ‘Left with a Sisyphean task’, reflects the nurses’ feeling of being handed the responsibility for performing a laborious, never-ending, and futile task.

**Conclusion:**

The findings indicate that nurse practitioners feel confident and competent in treating physical injuries due to NSSI but insecure about how to provide adequate care. Therefore, there is a need for training and guidelines.

**Supplementary Information:**

The online version contains supplementary material available at 10.1186/s12873-023-00888-6.

## Background

Non-suicidal self-injury (NSSI), defined as intentional self-inflicted damage to the surface of the body, such as cutting, burning, or hitting, performed without suicidal intent [1], is a prevalent phenomenon that especially affects adolescents and young adults [2, 3]. While an overlap between suicidality and NSSI exists, preliminary findings indicate that NSSI should be recognized as a distinct syndrome [4]. This differentiation sets it apart from borderline personality disorder and from suicide attempts [4]. Lifetime prevalence of NSSI is an estimated 18% for adolescents [5].

NSSI represents a significant health concern as it is linked to an increased risk of repeated NSSI, suicidal behaviour, and suicide, where individuals who frequently self-injure and use multiple methods are at the highest risk of committing suicide [2, 6, 7]. However, the predictive value of NSSI for suicide is unknown since estimates are confounded by suicidal self-injurious behaviour [8, 9]. Previous research shows a 68% increased likelihood of committing suicide for people with a history of deliberate self-harm, a definition of self-injury that includes suicidal self-injury compared to people without a history of deliberate self-harm [8]. Furthermore, the cost of hospital care due to self-injury is increasingly high [10].

Self-injuries are frequently seen in somatic emergency departments (EDs) [10]. For people with self-injuries, EDs are often their first point of contact with the healthcare system [11], with ED nurses being the most consistent group of healthcare professionals who treat them [11, 12]. Previous research on the perspectives of service users toward the treatment provided in EDs shows that people with self-injurious behaviour try to limit their contact with ED services based on their own negative ED experiences or that of peers [13]. Negative treatment experiences, such as being denied information, excluded from decision making, talked about as if not present, or ignored while having the injury treated in silence led to avoidance in seeking help in the future and possibly adverse health outcomes since participants stated that they were more likely to repeat self-injury following negative ED experiences [13].

Despite the important role of nurses in the treatment of self-injuries in EDs, a literature search of previous research exploring their experiences with treating people with NSSI in the ED only found studies that use a definition that includes self-harm, irrespective of suicide intent, and primarily surveys on the attitudes of ED nurses toward people who self-injure [12, 14–17]. These studies indicate the prevalence of certain attitudes but do not offer an understanding of the attitudes and their subtleties, and therefore only enable recommendations for practice to a limited extend. A recent qualitative study done in Australia [18] examined the experiences of ED nurses working with people who self-harm, including self-harm irrespective of suicide intent, also including experiences with people who had, e.g. taken an overdose. The present study aimed to explore the experiences of ED nurses specifically in relation to NSSI and aspired to include nurse practitioners qualified to treat injuries such as wounds due to cutting. The aim of this study was to describe the experiences of nurse practitioners with treating people presenting with NSSI in the ED.

## Methods

### Study design

This study employed a descriptive qualitative design. We generated data from individual, semi-structured telephone interviews, and used an inductive approach to content analysis.

### Study setting

The study took place in three EDs at three university hospitals in the Capital Region of Denmark. Two EDs are open 24 h a day, each serving approximately 80,000 patients per year and employing about 80 nurses. The third ED operates during daytime hours, serving approximately 40,000 patients per year and employing around 30 nurses. These three EDs cover distinct catchment areas, each with significantly varying social demographics in and around the capital of Denmark.

### Sampling method and participants

Due to the COVID-19 pandemic sampling and recruitment of participants was carried out by telephone and email. To ensure a broad representation of various characteristics that may influence the experience of the phenomena under study, purposeful sampling was used [19]. Thus, we recruited nurse practitioners, i.e. nurses with a specialist training in ED nursing practice, of various ages, sex, years as a registered nurse, years as an ED nurse practitioner and years of ED employment and who had any experience in treating people with NSSI.

**Fig. 1:**
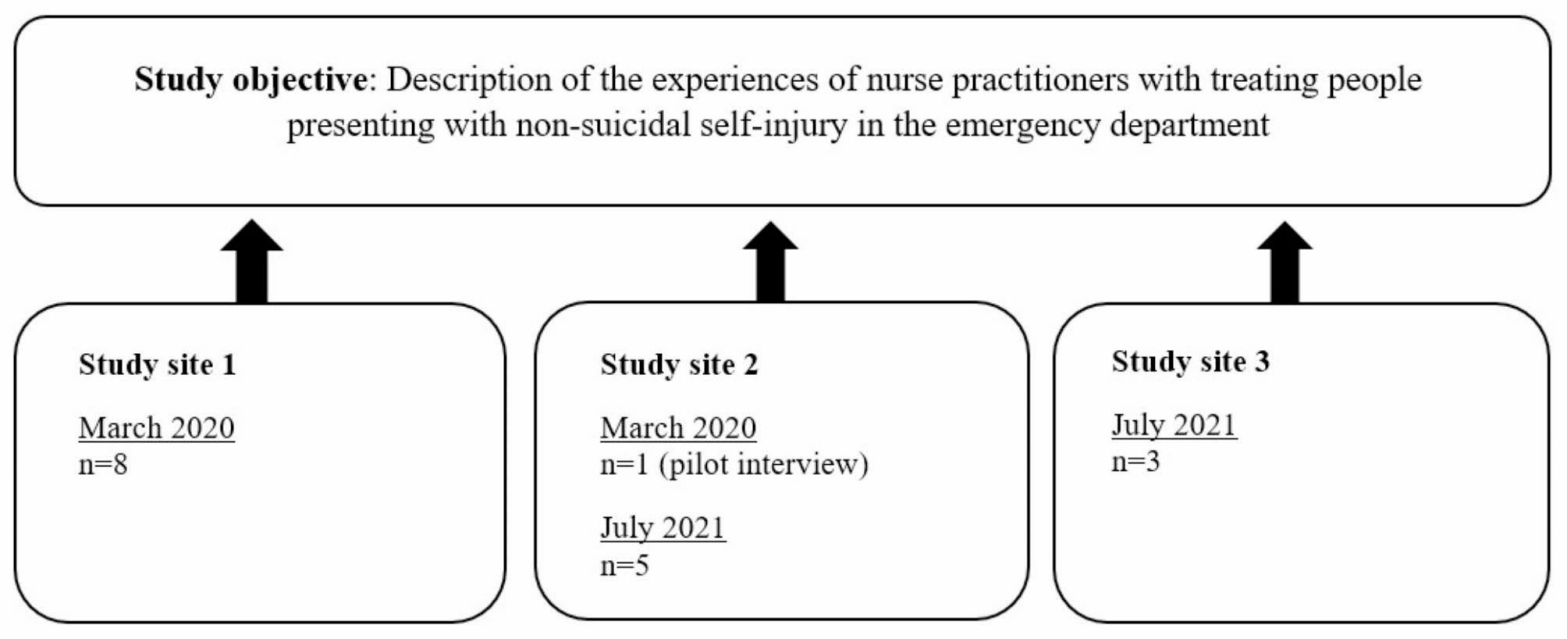
Overview of the recruitment of participants

The second author (CB) contacted the ED nurse manager at site 1 and the first author (KR) contacted them at sites 2 and 3 to ask if they would agree to participate in the study and to act as gatekeepers in the recruitment process. All three agreed to participate. The nurse managers were asked to identify nurse practitioners in their EDs based on the sampling criteria and to provide the email addresses of anyone interested in participating. Those who showed interest (n = 17) were contacted via email containing information about the nature of the study and a request to express their willingness to participate. All 17 individuals agreed to participate and subsequently received an email with a declaration of consent and were asked to find a convenient time for them to participate in a telephone interview. Figure 1 provides an overview of the recruitment process for participants.

### Interviews

Individual, semi-structured telephone interviews were used to generate data to gain insight into the experiences of the individual nurse practitioner [20]. The interview guide and interviews used in this study was developed for this study and have not previously been published. The interview guide was developed based on the study aim, current scientific literature, and feedback from a nurse practitioner at study site 2. According to Crabtree and Miller [20], the interview guide consisted of both closed-ended, identifying questions and broad, open-ended questions with accompanying prompts and probes, e.g. “How does this influence you?” or “Can you tell me more about that?” to ensure data rich in detail, depth and subtleties (see Additional file 1). CB conducted a pilot interview before discussing the applicability of the interview guide with KR. After only one minor adjustment to the interview guide (a question on the physical environment in the EDs was added as the pilot interviewee brought up this topic) the pilot interview was included in the data analysis.

Participants were told that the interview would take place without prejudice in that the researchers aimed to achieve a clear, in-depth and non-judgemental understanding of how the participants experience treating people with NSSI [21]. During the interviews. dialogic validation was employed as a technique to enhance intersubjectivity between the interviewer and the participant [22]. This served the purpose of elucidating answers, fostering mutual understanding, and validate the interview [22]. In essence, the objective was to eliminate potential misunderstandings, thus creating an optimal foundation for asserting that the analysis results captured the participants’ experiences [22]. Notes were taken during data generation related to the interview and to facilitate recall [20]. Each interview ended with a short interview on demographic data to enhance transferability [23].

### Reflexivity and trustworthiness

To ensure reflexivity and trustworthiness, authors involved in data generation and the initial analysis (i.e. KR, CB, and the third author (SY)) conducted an introspection of preunderstandings prior to designing the study and the interview guide. Specifically, we interviewed one another using a structured interview guide to create a declaration of beliefs [19]. This provided insight into our preunderstandings of not only people who engage in NSSI but also ED treatment of NSSI, ED nurse practitioners, our motivation for conducting the study, possible hypotheses on study findings, potential effects due to our backgrounds. KR is a registered mental health nurse and SY is a registered nurse, both with insider knowledge about working with people engaging in NSSI in inpatient mental healthcare settings, while CB is a registered physiotherapist with work experience in relation to one of the ED settings of the study (i.e. as a staff physiotherapist). None of us are ED nurses, however, and none of us has worked with treating the target group together with the participants. To ensure credibility, an audit trail was kept throughout the study process [23].

### Analysis

The study employed a descriptive qualitative approach to data analysis [24]. Content analysis, as described by Graneheim and Lundman [25] was chosen based on the study objective of examining the sensitive topic of the actual experiences of nurse practitioners treating people with NSSI in the ED. Based on limited scientific knowledge about the phenomenon, an inductive approach to content analysis was called for [24].

All interviews were audio-taped and transcribed verbatim. The interviewers transcribed 14 interviews and a research assistant transcribed the other three. To ensure consistency in the translation from oral to written discourse, a transcription manual with transcription conventions was applied [21]. To support the reliability and accuracy of the transcripts, KR and CB both separately transcribed the first five minutes of the first nine interviews [21]. The transcriptions were compared, and any discrepancies were marked and discussed. As the discrepancies only reflected minor differences (e.g. addition of an extra word to a sentence), the rest of the transcripts were verified by comparing the transcript to the audio file [21].

First, the analysis began by having two authors read and reread the transcriptions to obtain an overall understanding of the content of the data. Next, the text was divided into meaning units corresponding to words, sentences or paragraphs related to the study objective. The units of meaning were then condensed before being compared and coded. During the first phase, the analysis the researchers doing the coding each analysed two interviews separately. The tentative results of the analysis were compared and discussed. A small difference was detected in the division of the units of meaning, their condensed meaning, and corresponding codes. The remainder of the data material was analysed jointly. All condensed units of meaning were compared, discussed, and coded. The next step involved abstracting, comparing, and sorting the codes into 10 subcategories, which were then used to create three categories based on the similarities and differences between the subcategories. Before proceeding, a third author read two interviews and took part in discussing the results of the analysis. As the data material allowed for a deeper interpretation of the underlying meaning of the categories and their subcategories, further abstraction led to the formulation of one overarching theme. Finally, to ensure consistency, the original transcripts were reread and compared against the overarching theme, categories, and subcategories that were generated.

Table 1 provides examples of the analytical condensation-abstraction process from units of meaning to categories. Data management was conducted using NVivo 12 (QRS International, Melbourne, Australia).

**Table 1:** Examples of the condensation-abstraction process for the category Groping in the dark

Unit of meaning	Condensed unit of meaning	Code	Subcategory	Category
*Example 1* How do I best talk to them without making it worse for them? Is it best that I ask directly or should I be more careful? (NP13)	Questions how to converse beneficially with patients about NSSI	Insecurities regarding how to converse about NSSI with patients	Staring into an empty toolbox	Groping in the dark
*Example 2* I think that if you’re sad and frustrated, then you wouldn’t have the energy to do a full face make-up (NP2)	Factors that contradict one’s understanding of patients with NSSI	Trying to understand NSSI and its functions	Trying to grasp NSSI	

*NP* Nurse practitioner, *NSSI* Non-suicidal self-injury

## Results

### Sample characteristics

Seventeen nurse practitioners participated in the study. Interviews lasted 34 min on average, the shortest interview lasting 20 min and the longest 50 min. Ten participants were interviewed while at work and seven while at home. Table 2 presents the demographic characteristics of the participants.

**Table 2:** Participant characteristics (n = 17)

Characteristics	n (unless stated otherwise)
Nurse practitioner	
Study site 1	8
Study site 2	6
Study site 3	3
Age, mean in years (min–max)	45.6 (26–65)
Sex	
Female	17
Male	2
Years as a registered nurse, mean in years (min–max)	16.9 (3–40)
Years as emergency nurse practitioner, mean in years (min–max)	8.1 (2–24)
Years of ED employment, mean in years (min–max)	13.2 (3–38)

*ED* Emergency department

### Content

The content analysis resulted in the formulation of one overarching theme and three categories covering 10 subcategories (Table 3).

**Table 3:** Results of the content analysis

Overarching theme	Category	Subcategories
Left with a Sisyphean task	Groping in the dark	Staring into an empty toolbox Relying on your own standard procedures Trying to grasp NSSI
	An unrewarding and burdensome task	From investment to powerlessness and aversion Witnessing ill-treatment Worrying about patients’ wellbeing after discharge
	Systemic and societal failures	A divided healthcare system Absence of NSSI-specific guidelines and training in the ED Lack of resources in the mental healthcare system Society is failing people with NSSI

*NSSI* Non-suicidal self-injury, *ED* Emergency department

### Groping in the dark

This category (including 3 subcategories) refers to the nurse practitioners’ experience of an absence of direction and lack of NSSI specific training, guidance, and guidelines contributing to nurse practitioners’ feeling of uncertainty and insecurity.

#### Staring into an empty toolbox

The nurse practitioners described themselves as confident and competent in managing treatment of the physical injuries from NSSI and took pride in their skills. As opposed to being specialised and guided by clinical guidelines in the treatment of physical injuries, the nurse practitioners painted a portrait in which they were groping in the dark in terms of NSSI and as lacking specific training and guidelines to rely on. They described themselves as lacking in mental healthcare competences, insecure and as self-conscious during interactions with patients, just as they were concerned that they might do more harm than good:

*“I’d feel really bad if they went away feeling they’d had a terrible experience and then slid further into their depression, or whatever.”* (Nurse practitioner (NP) 11).

The nurse practitioners refrained from engaging in conversations about NSSI and mental wellbeing because they were unsure about what the conversations would consist of and whether they benefited patients:

*“I’ve no clue where I should start and end, nor do I know what she needs, so I sometimes think ‘Does she just need to get it fixed and then go home?’”* (NP9).

Some stated that encounters with patients with NSSI included an assessment of suicide risk, which they described as feeling somewhat confident about doing. However, at the same time, they questioned whether they, in the absence of instructions to guide them, were able to correctly identify suicidal patients:

*“That is, if it [the wound] is really deep and really big, then I have to get a doctor involved, who, in that case, must decide: are we talking involuntary hospitalisation or what should happen? It’s a very difficult assessment that I have to make based a bit on how I feel. What’s my gut feeling about what this is.”* (NP17).

#### Relying on your own standard procedures

Having no specific training or guidelines to inform their practices led to large variations in the treatment offered to patients with NSSI:

*“Our group of staff is very diverse, and we have highly different approaches to treating the self-injuring girls that come to us.”* (NP3).

The nurse practitioners recounted how they were forced to rely solely on their extensive ED experience, including developing their own standard procedures or copying their colleagues’ approaches to NSSI. Having seen many patients repeatedly return to the ED with NSSI, a group referred to as frequent flyers^1^, some nurse practitioners described using preventive strategies that they had invented to hinder repeated NSSI. Strategies included encouraging patients to seek or sustain mental healthcare; use coping strategies such as calling a friend; negotiating; normalising what the patient was dealing with by stressing that youth is often a troubling phase; or by talking to patients about the possible physical or social consequences of their behaviour:

*“‘You see the ugly arms you get; you’ll have to live with this the rest of your life’. I can say this several times, and I’m also thinking, ‘Well, it doesn’t help’ but I just feel that I need to say it.”* (NP17).

The nurse practitioners described how rewarding it was when patients who had recently begun injuring themselves, a group referred to as beginners, accepted their advice to seek further help. However, they questioned the effectiveness of their preventive strategies and requested evidence-based preventive tools.

#### Trying to grasp NSSI

A common feature of the interviews was that the nurse practitioners related NSSI to mental health and portrayed it as a maladaptive coping strategy for reducing mental distress or as a cry for help due to a failure to thrive. However, the nurse practitioners stated that factors such as being nicely dressed, wearing full make-up, having perfectly painted nails, appearing to be indifferent toward their injuries or being in a good mood contradicted their understanding of patients with NSSI:

*“Pretty good mood sometimes, amazingly good mood; they make jokes with me, and they make jokes with the staff. Sometimes you may think that they take it exactly as, well, a cosy little trip to the emergency department. Well, I get certain thoughts about it, I really do.”* (NP17).

This type of behaviour and appearance made the nurses perceive some patients with NSSI as young adults engaging in a mainstream, yet destructive, youth culture fuelled by its presence on social media. A tendency to view the severity of the physical injury as a predictor for what was described as a true NSSI issue was also a topic that was touched upon across interviews. Patients with deep or multiple cuts were perceived as having a true NSSI issue and advised to seek mental healthcare, whereas patients with superficial injuries were judged as not having a need for mental healthcare. When men presented with self-inflicted fractures to the hand, the nurse practitioners described their behaviour as a quick fix led on by immediate frustrations rather than an action reflecting underlying mental issues and hence deemed that there was no need for mental healthcare. NSSI was depicted as attention-seeking behaviour which, at one research site, was exemplified by a case of a patient with borderline personality disorder whose frequent visits to the ED due to NSSI had been time-consuming and involved multiple staff and the use of coercion. The ED staff had consequently been advised to limit the amount of attention given to the patient. Accordingly, nurse practitioners from the site described that attention should be limited for all patients with NSSI and/or borderline personality disorder to hinder repeated NSSI and visits to the ED:

*“That’s all they really want to achieve, and the more they will come, and the more often they will hurt themselves, those with borderline too, the more attention they get.”* (NP2).

### An unrewarding and burdensome task

This category encompasses 3 subcategories related to the nurse practitioners’ experiences of the treatment of NSSI as unrewarding. This category, moreover, highlights an emotional shift among nurse practitioners over time, from initial investment to feelings of powerlessness and aversion.

#### From investment to powerlessness and aversion

Upon first encountering NSSI, the nurse practitioners described a desire to educate themselves on NSSI and to find ways to secure help for people with NSSI. However, over time, increased familiarity led to the treatment of it becoming just another routine task. The nurse practitioners depicted treatment as time-consuming and the self-inflicted nature of NSSI was portrayed in stark contrast to other ED tasks, which gave rise to resistance towards patients. Helping someone with NSSI was regarded as less fulfilling and rewarding than treating people who had unwillingly got injured:

“*You may look at it [the wound] and say ‘Well, that turned out nicely’, but [laughs] you also just know that it’ll just be another scar in a row of, well, uh … So, you don’t feel the same sense of satisfaction as you do when you have stitched up someone with something that happened by accident. I might have patched her together, but it hasn’t solved ANYTHING at all.*” (NP9).

Numerous encounters within short periods of time lead to feelings such as exhaustion, powerlessness, frustration, hopelessness, and irritability. The nurse practitioners described exercising restraint in showing negative feelings toward patients, but also communicated that this strategy can become difficult when encounters accumulate:

“*But sometimes you can hardly control yourself. Because you can get tired of it if it’s the same person who shows up twice in one shift. I will honestly admit that once, I simply told someone that I didn’t want to see her once again, which was, of course, a bit harsh. I felt really bad about that, but on the other hand, then sometimes, you simply cannot continue to accommodate everything.*” (NP15).

Some nurse practitioners stated that they were embarrassed to admit that they perceive patients with NSSI as difficult. Others stated a more direct aversion towards frequent flyers and spoke of a drama that they tried not to get caught up in:

“*One must be careful not to show them too much pity, because they will feed on it. Then there’s the gotcha moment and you’re stuck [laughs] … these are some difficult patients, let’s just put it that way.*” (NP7).

#### Witnessing ill-treatment

Treatment of NSSI was depicted as causing feelings of distress when nurse practitioners witnessed what they described as certain colleagues handling treatment poorly:

“*I get a big knot in my stomach if it’s one of them who gets this kind of patient. I can’t stand it. I can get really upset.*” (NP14).

Nurse practitioners provided various examples of overhearing ill-treatment by colleagues, which included shaming patients for their actions, denying them anaesthetics by claiming they enjoyed the pain or by objectifying them as ideal cases for practicing suturing skills:

*“‘They don’t notice anything anyway’ and ‘You can just stitch up the wound without giving them an anaesthetic’ and ‘Their wounds are straight lines, they’re really easy to stitch.’”* (NP6).

The nurse practitioners emphasised that this did not prompt them to alter their own practices. On the contrary, they felt assured that what they described as ill-treatment did not contribute to patients’ wellbeing. Consequently, they preferred to personally treat all patients with NSSI rather than having their colleagues handle the treatment inadequately.

#### Worrying about patients’ wellbeing after discharge

Some nurse practitioners perceived the ability to make it to the ED as a sign that patients were able to take care of themselves, while others feared that patients might continue to self-injure or commit suicide if they left the ED without seeking mental healthcare:

*“You know, your worst fear is sending a person home and finding out the next day that they’ve jumped off a tall building.”* (NP11).

Such concerns were connected to feelings of powerlessness, which could only be quelled if patients were discharged to ongoing treatment at a mental health unit or residential mental health institution.

### Systemic and societal failures

This category (including 4 subcategories) depicts the nurse practitioners’ disappointment and disillusionment with the healthcare system’s limitations, including a lack of resources, inability to provide adequate care, and a perceived societal failure to address the underlying causes of NSSI.

#### A divided healthcare system

The nurse practitioners often referred to the organisational divide between mental and physical healthcare when describing their main area of responsibility as taking care of physical health:

*“We’re used to treating wounds, suturing, and bandaging and all the rest; we do it day in day out. But it’s the specialists who take care of the mental health side of things.”* (NP5).

They often made it clear to patients that the somatic ED exclusively specialises in physical injuries, whereas the mental health unit provides care for mental health issues related to NSSI. During interviews, they nonetheless also described how mental healthcare services distinguish between suicidal self-injury and NNSI when considering a person’s need for mental healthcare, which is why mental health care may not be provided for all patients with self-injury:

*“Sometimes it rings a bit hollow when you say that you can just send them on to the mental health unit, or that it’s a service we offer. And then, too, there’s the fact that I don’t really know what else to offer them.”* (NP13).

In virtue of this, the nurse practitioners depicted people with NSSI as marginalised by the healthcare system, making it seem like no one really cares about what they are facing:

*“If their life is not in danger or they are not a danger to themselves, it can be pretty hard to find immediate help for them.”* (NP8).

#### Absence of NSSI-specific guidelines and training in the ED

Due to an absence of NSSI-specific guidelines and training in the ED, the nurse practitioners requested specialised, evidence-based knowledge and NSSI-specific guidelines to improve the quality of care, standardise the treatment and help alleviate their insecurities:

“*It would be really, really nice for many of us to have greater competence in this, and I really think it would ease some situations if we had some evidence, or a knowledge base, to support why and what we can do, or how we could do things differently.*” (NP13).

The nurse practitioners requested short educational sessions or one-day seminars on NSSI. They stated a need for clinical guidelines and a semi-structured questionnaire of what to ask patients, including signs to look out for hindering misinterpreting patient needs and the risk of suicide. Further, they requested information on where to refer patients and greater collaboration with mental healthcare units to build a mutual understanding of work responsibilities and services offered in both entities.

#### Lack of resources in the mental healthcare system

Frustrations related to a failing healthcare system were amplified when patients are residents at a community mental health institution or inpatients at a mental healthcare facility, leading to a distrust in the ability of these entities to adequately care for patients with NSSI:

*“I think there’s something fundamentally wrong about the system because we’re giving the patients sub-optimal help. It’s very frustrating, I mean I’m prepared to ring up if someone is hospitalised [in a mental healthcare unit] and enquire. It’s just not right.” (NP10)*.

Concurrently, the nurse practitioners acknowledged the limited capacity in mental healthcare services and called for an increase in both the resources and quality of care provided in mental health facilities, as well as additional treatment options for patients who are not in immediate danger of suicide.

#### Society is failing people with NSSI

Nurse practitioners criticised what they perceived as a limited commitment by society to treat and prevent repeated NSSI and demanded sufficient and adequate treatment options for people with NSSI. As a result, some described themselves as feeling disillusioned in their role in treating NSSI:

*“Sometimes you may feel ‘Nooo, she can’t be here AGAIN’, because it’s so frustrating. It seems so useless to just sit there and patch people together, and then you can remove the sutures you stitched up the week before.“* (NP9).

Moreover, they found it maddening to witness endless growth in the prevalence of NSSI and called for action to ensure primary prevention efforts targeting the underlying causes of NSSI at the societal level:

*“We [society] must be failing somewhere since we have so many of them. Something must have been done wrong or is being done wrong.”* (NP12).

### Left with a Sisyphean task

In the last phase of the analysis, the underlying meaning of the experiences of nurse practitioners with treatment of people with NSSI in the ED was interpreted and formulated into the overarching theme ‘Left with a Sisyphean task’. A Sisyphean task symbolises the laboriousness of performing a never-ending, futile task that may potentially lead to feelings of resignation, numbness and disbelief [27]. The results of this study suggest that the nurse practitioners experience inter- and intraindividual disagreements, insecurities, and difficulties concerning how to manage NSSI due to the lack of clarity surrounding how to conceptualise NSSI and its functions. Their experiences point to a system failure in which society and the mental healthcare system, including community-based services, inadequately meet the needs of people with NSSI. This situation leaves nurse practitioners to patch up the wounds bereft of tools to stop the predicament and without recognition of the problem. Like with a Sisyphean task, the nurse practitioners experience treating NSSI as meaningless and as an impossible task to complete.

## Discussion

In this study, which aimed to describe the experiences of nurse practitioners with treating people presenting with NSSI in the ED, we found that nurse practitioners felt powerless in the face of NSSI. Nurses described being able to treat only the wound and were disappointed with the inability of mental healthcare services to prevent repeated NSSI and provide adequate treatment options, which led us to the depiction of treatment of NSSI as a Sisyphean task. Hadfield et al. [28] similarly show that ED doctors view treating people with self-injuries as a futile task that causes feelings of despair and frustration. The nurse practitioners in the present study also felt powerless about the situation, since they felt like the system was not geared or committed to securing adequate and skilled treatment for the factors underlying the behaviour. They pointed to a larger systemic failure in which society is to blame for failing to act against the growing incidence of NSSI, as well as its inability to provide effective treatment options.

Overall, our findings suggest that nurse practitioners feel competent and take pride in treating physical injuries resulting from NSSI. However, the findings also suggest that nurse practitioners may perceive their duty of care as restricted to involving phenomena understood within the traditional biomedical model. Results from a systematic review by Taylor et al. [29] on attitudes toward clinical services among people with NSSI found that the pronounced focus on physical injury in ED settings is experienced negatively by people seeking treatment for self-injury, who perceive ED staff as being unconcerned with their mental wellbeing. Nurse practitioners in the present study viewed the context (i.e. somatic EDs) and the absence of NSSI specific guidelines as legitimising a strictly limited focus on wound care. This stands in contrast to the internationally widespread and contemporary person-centred approach to policy and delivery of healthcare [30], which is also reflected in Danish healthcare policies [31]. In addition to ensuring the physical needs of patients, the approach aims to encourage shared decision making and the integration of the individual’s values and concerns into the treatment being delivered, a strategy that has been shown to increase patient satisfaction with treatment [30]. In an effort to compensate for a limited understanding of NSSI, the nurse practitioners in the present study requested NSSI-specific training and guidelines to inform their decision making. McAllister et al. [32], who examined the effectiveness of an educational intervention directed at the responses of ED nurses toward people who self-harm, showed that the nurses obtained skills that helped them cope with, respond to and engage in conversations with people who self-harm [32]. Future research should investigate whether NSSI-specific guidelines can also serve to strengthen ED nurse practitioners’ perceived skills toward managing treatment and engaging in conversations with people with NSSI.

A review by the National Institute for Health and Care Excellence in the UK [33] underpins the suggestion put forward by the nurse practitioners in our study that alternative strategies can be applied to cope with negative feelings and to distract thoughts of NSSI. The review showed that employing alternative strategies, such as verbalising emotions and reaching out to others, is crucial to stopping self-injurious behaviours [33]. The present study identified a trivialisation of the underlying reasons why men engage in NSSI. However, qualitative research show that for men self-injury is not exclusively a quick fix to momentary frustrations, rather it can provide several days’ relief from mental distress [34]. Of further interest, the study found that shame hindered help-seeking in men who wished to stop self-injuring [34]. This adds to the understanding that men engaging in NSSI are overlooked by healthcare professionals, which might subsequently hinder prevention of repeated NSSI in men [35]. Hence, future NSSI guidelines should also specifically focus on how to promote the prevention of self-injury in men.

A notable finding in this study was that the nurse practitioners tended to appraise the severity of the injury and the patients’ appearances to determine the validity of NSSI as a genuine issue. These findings indicate that merely presenting with wounds due to NSSI does not guarantee everyone with NSSI will be seen as equally deserving and in need of care. In a Danish study exploring how discourses on mental illness are negotiated in mental health practice, Ringer and Holen [36] found that conceptualisations of mental illness position people in specific ways, forcing them to navigate how to come across and appear accordingly during interactions with healthcare professionals. Their study identified the discourse of being “really ill” to illustrate an understanding in which people can be perceived as either “authentically” or “inauthentically” ill, which in return requires people to present themselves with more than the appropriate symptoms of a specific diagnosis to convincingly be acknowledged as really ill, and thus easier to provide with care [36]. This points to a difficult balancing act that people must master to be regarded as deserving of care; hence, in their interactions with healthcare professionals, people can be “doing ill” wrongly [36]. For the nurse practitioners in our study authentically doing ill meant the signs of mental distress had to be somewhat unobtrusive, e.g. looking outwardly unhappy was acceptable but appearing either too well or too distressed less so, the latter leading to the use of coercion and spending much time in treatment.

Another noteworthy finding of this study was the possible failure to use anaesthetic during suturing. This finding is supported by previous studies, including a systematic review [13, 29] of ED service user experiences who had been denied pain medication or anaesthetics due to the self-inflicted nature of the injuries. The authors of the present study recommend future research to investigate how widespread the phenomenon is.

Our study also highlighted how nurse practitioners understood NSSI as attention-seeking behaviour. This is in line with multiple studies and theories on self-injurious behaviour [28, 33, 36, 37]. For example, Hadfield et al. [28] found that ED doctors perceive treatment of NSSI as exacerbating attention-seeking behaviour, which is why they refrained from giving too much attention to people with NSSI. A previous study explored service user experiences of ED treatment for self-injuries and revealed that when care providers withheld sufficient attention, it resulted in a tendency to avoid seeking help in the future [13]. Subsequent research needs to elucidate whether limiting the extent of attention provided to people with NSSI in the ED context deters future instances of NSSI or solely impacts visits to the ED and, in a broader sense, influences help-seeking behaviours. Other labels, such as frequent flyers and beginners, were also used to identify people with NSSI, which is in line with McGough et al. [18], who found that labelling people who frequently visit the ED occurs among healthcare staff. Labelling threatens to dehumanise people [38] and is known to facilitate stereotyping in clinical settings of people who struggle with mental health issues [26, 39]. Labels can carry harmful, implicit biases [26] that negatively affect clinical outcomes for the people they describe [26, 39]. Hasking and Boyes [38] assert that a lack of knowledge about NSSI is associated with the language people use to address NSSI and respond to people with NSSI. A useful path to pursue in future research on educational interventions is whether an improved understanding of NSSI results in an altered, less harmful rhetoric.

Although our study participants were informed about the definition of NSSI and asked to avoid referring to their experiences with treating suicidal self-injury, the study indicates that distinguishing between cases of suicidal and non-suicidal self-injury is difficult. They were aware that the mental healthcare unit distinguishes between the two phenomena when assessing the need for mental healthcare but this did not prevent them from making many references to suicidality and suicidal behaviour. In the same vein, the study identified a tendency to consider NSSI as a mental health issue that must be treated in mental healthcare services. While the prevalence of NSSI is higher in clinical samples than in the general population [2], people who engage in NSSI as a symptom of mental illness belong to the minority, as the majority are normally developing individuals who engage in NSSI in non-clinical settings [40]. McGough et al. [18] identified the same tendency, which points to a need for further study of self-injurious behaviours and a clarification concerning terminology, definition, and assessment methods.

### Methodological considerations

The purposeful sampling strategy provided a relevant sample that varied in terms of sex, age and years of ED experience, resulting in a thick description of the phenomenon under study. The three ED nurse managers whom we contacted to recruit nurse practitioners for our research readily allowed their participation in the study. However, it is worth noting that the involvement of gatekeepers, in this case nurse managers, during recruitment might have led to the exclusion of nurse practitioners holding negative attitudes towards NSSI from participating in the study. As a result, relying solely on training and guidelines might prove inadequate in enhancing the care provided to individuals with NSSI. Ideally, comprehensive competence development should encompass not only the acquisition of skills but also the cultivation of awareness regarding attitudes and the promotion of reflection on the nurses’ influence on the patients’ trajectory.

KR, CB and SY each conducted 8, 4 and 5 interviews, respectively. At the research site where CB worked as a staff physiotherapist, five interviews were carried out by KR, while CB interviewed 3 participants whom she had not threated. The participation of all authors in the analysis adds to the credibility of our study [41]. Triangulation of sites further increased the credibility since the representation of participants from different hospitals reduced the risk of findings resulting from factors peculiar to one site [23]. Further, a detailed description of the study setting and participants support the transferability of our findings to local settings [23].

Face-to-face interviews are regarded as the gold standard of data generation in qualitative inquiries, but the assumption is that the quality of the data, and thus the research findings, are somewhat compromised in telephone interviews due to factors such as a lack of visual cues [42]. Telephone-based data generation does have some beneficial qualities [42]. In our study, for example, participants could remain on their own turf and to talk freely about sensitive information [42], which supported the generation of rich data and dense descriptions. Using only a single mode of data generation (i.e. individual interviews) can be considered a limitation because we did not have direct access to their actions and behaviour in clinical practice. Thus, a possible avenue for future research is carrying out an ethnographic inquiry.

## Conclusion

In conclusion, this study found that the experiences of nurse practitioners with treating people with NSSI in the ED showed that they viewed treatment as a Sisyphean task, in other words, as laborious and futile. The findings indicate that nurse practitioners feel confident and competent in treating physical injuries due to NSSI but insecure about how to provide adequate care and engage in conversations about NSSI and mental wellbeing with people with NSSI. The findings further indicate that not all people presenting with NSSI in EDs are considered equally deserving and in need of care, as some people are viewed as performing NSSI untruthfully or as appearing either too well or too distressed. Hence, providing nurse practitioners with NSSI-specific training and guidelines to direct their decision making and strengthen their confidence in their interactions with people with NSSI appears warranted.

### Electronic supplementary material

Below is the link to the electronic supplementary material.

Supplementary Material 1

## Data Availability

Please contact the first author, Kickan Roed, to request data (kickan.roed@regionh.dk).

## Abbreviations

ED: Emergency department
NP: Nurse practitioner
NSSI: Non-suicidal self-injury

## Footnotes

1 ED staff use the label frequent flyers colloquially when referring to a subpopulation of individuals with mental health conditions who frequently visit EDs [26, p. 1539]. Well-known by ED staff, frequent flyers are often viewed as super utilisers and problem patients [26, p. 1539].
