# Supplementary material for: Left with a Sisyphean task – the experiences of nurse practitioners with treating non-suicidal self-injury in the emergency department: a descriptive qualitative study
Source: BMC Emerg Med. 2023 Oct 5;23:117. doi: 10.1186/s12873-023-00888-6 (PMC10557284; doi:10.1186/s12873-023-00888-6)
Supplement: Supplementary file 1 — Supplementary Material 1 [file 12873_2023_888_MOESM1_ESM.docx]

*Left with a Sisyphean task – the experiences of nurse practitioners with treating non-suicidal self-injury in the emergency department: a descriptive qualitative study*

Kickan Roed^1^*, Cecilie Rostrup Brauner^1^, Senayt Yigzaw^1^, Julie Midtgaard^1,2^

* Correspondence: kickan.roed@regionh.dk

Mental Health Center Glostrup, Copenhagen University Hospital – Mental Health Services CPH, Centre for Applied Research in Mental Health Care, Nordstjernevej 41, 2600 Copenhagen Ø, Denmark.

^2^ Department of Clinical Medicine, Faculty of Health and Medical Sciences, University of Copenhagen, Blegdamsvej 3B, 2200 Copenhagen N, Denmark

**Additional file 1**

**Interview guide**

1. How often do you treat patients who have self-injured?

Follow-up questions:

- What types of injuries are these?
- What kind of treatment did these injuries require?

1. Can you describe a situation in which you treated a patient who came to the emergency department because of injuries caused by self-injury?
2. How do you care for and treat a patient with self-injuries?

Follow-up questions:

- What guides you when deciding on how to care for and treat a patient with self-injuries? Meaning guidelines, knowledge, skills you draw on.
- What are your ideals for how patients with self-injuries should be treated?

1. Which immediate thoughts go through your head when you find out that you must treat a patient who has intentionally harmed themselves?
2. Which immediate thoughts go through your head when you find out that you must treat a patient who regularly presents themselves with self-injuries in the emergency department?
3. Can you describe your experiences of collaborating with external partners, including mental health staff and patients’ relatives?

Follow-up questions:

- What challenges does this present?
- What advantages/disadvantages have you experienced regarding collaboration with external partners?

1. To what extent do you feel competent/qualified to handle the treatment of self-injuries?

Follow-up questions:

- Have you thought about and/or have you talked to your colleagues about how you might improve your skills in order to handle this task?
- What is your biggest asset in relation to the treatment of this patient group and where are your biggest challenges/shortcomings?
- Is there anything you lack or could wish for that could contribute to making your work with these patients better for you and for the patients?
- How do you experience the physical environment of the emergency department in relation to the treatment of self-injuries?

1. How do you handle registration of self-injuries in the patient record?
2. How do you experience discharging patients after having treated them for self-injuries?

Follow-up question:

- What are your thoughts on discharging patients after they have been treated for self-injuries?

Prompts to use throughout the interview:

- How does this influence you?
- What impression does this leave on you?
- How does it affect you?
- What thoughts are going through your head?
- Is this the only time you have been in this situation?
- Can you tell me something more about that?
- Can you elaborate on that?

Closing the interview:

- That was the last question I had; do you have anything you would like to add about your experience of treating patients with self-injuries?
- How was it for you to take part in the interview?
- May I ask you why you have chosen to participate in the interview?

Demographic questionnaire:

- How old are you?
- How long have you been a registered nurse?
- How long have you been trained as an emergency nurse practitioner?
- How long have you worked in an emergency department?
